# Anti-Osteoporotic Effects of Combined Extract of *Lycii Radicis Cortex* and *Achyranthes japonica* in Osteoblast and Osteoclast Cells and Ovariectomized Mice

**DOI:** 10.3390/nu11112716

**Published:** 2019-11-09

**Authors:** Eunkuk Park, Jeonghyun Kim, Subin Yeo, Eunguk Lim, Chun Whan Choi, Sangho Choi, Wan Yi Li, Ji-Won Lee, Jin-Hyok Park, Dam Huh, Seon-Yong Jeong

**Affiliations:** 1 Department of Medical Genetics, Ajou University School of Medicine, Suwon 16499, Korea; jude0815@hotmail.com (E.P.); danbi37kjh@hanmail.net (J.K.); eunguk@ajou.ac.kr (E.L.); 2 Department of Biomedical Sciences, Ajou University Graduate School of Medicine, Suwon 16499, Korea; 3 Nine B Company, Daejeon 34121, Korea; snsnans@naver.com; 4 Natural Products Research Institute, Gyeonggi Institute of Science & Technology Promotion, Suwon 16229, Korea; cwchoi78@gmail.com; 5 International Biological Material Research Center, Korea Research Institute of Bioscience and Biotechnology, Daejeon 34141, Korea; decoy0@kribb.re.kr; 6 Institute of Medicinal Plants, Yunnan Academy of Agricultural Sciences, Kunming 650200, China; wyli2012@126.com; 7 Korea Food Research Institute, Seongnam 13539, Korea; dnjs0004@naver.com; 8 Dongwoodang Pharmacy Co., Ltd., Yeongchen 38819, Korea; navy9376@hanmail.net

**Keywords:** *Lycii Radicis Cortex*, Achyranthes japonica, osteoporosis, ovariectomized mice, osteoblast, osteoclast

## Abstract

Osteoporosis is characterized by low bone density and quality with high risk of bone fracture. Here, we investigated anti-osteoporotic effects of natural plants (*Lycii Radicis Cortex* (LRC) and *Achyranthes japonica* (AJ)) in osteoblast and osteoclast cells in vitro and ovariectomized mice in vivo. Combined LRC and AJ enhanced osteoblast differentiation and mineralized bone-forming osteoblasts by the up-regulation of bone metabolic markers (*Alpl, Runx2* and *Bglap*) in the osteoblastic cell line MC3T3-E1. However, LRC and AJ inhibited osteoclast differentiation of monocytes isolated from mouse bone marrow. In vivo experiments showed that treatment of LRC+AJ extract prevented OVX-induced trabecular bone loss and osteoclastogenesis in an osteoporotic animal model. These results suggest that LRC+AJ extract may be a good therapeutic agent for the treatment and prevention of osteoporotic bone loss.

## 1. Introduction

Bone is continuously remodeled and maintained by a balanced process between bone formation and resorption [1,2]. Bone metabolism is a physiological condition that is controlled by depositing new bone formation by osteoblasts and resorbing old or damaged tissue by osteoclasts [1]. Osteoblasts are specialized mesenchymal stem cells responsible for the synthesis and mineralization of bone formation. Osteoclasts break down or dissolve bone tissue and play a central role in the maintenance and remodeling of bone from vertebral skeleton. An imbalanced relationship between bone resorption and formation leads to inappropriate bone remodeling, resulting in serious bone loss with bone metabolic diseases such as osteoporosis and osteopenia [3].

Osteoporosis is characterized by low bone density and quality with microarchitectural disruption [4]. Osteoporosis in elderly or postmenopausal women is associated with aggressive bone resorption, leading to an enhanced risk of bone fragility and susceptibility to fractures [5]. Recently, various pharmacological osteoporosis therapies have been discovered to reduce fracture risks, such as inhibitors of bone resorption (bisphosphonates) or activators of bone formation (*parathyroid hormone* analogs) [6]. However, some of these agents have limitations for long term treatment due to side effects such as increased risk of endometrial and breast cancers [7].

Recently, benefits of natural plants have revealed alternative modern therapies for the treatment of many diseases, including fewer side effects and suitability for long-term use [8,9,10]. *Lycii radicis cortex* (LRC), Lycium Chinese root bark, and *Achyrantes japonica* (AJ), Japanese Chaff flower, have been widely used in eastern Asia as traditional medicine. We previously screened 64 ethanol extracts of edible plants native to Korea for their ability to increase the cellular proliferation and differentiation of osteoblastic cell line (MC3T3-E1) and found the LRC extract as a best candidate for osteoblast differentiation. The study reported that LRC extract inhibited bone mineral density (BMD) loss in an ovariectomized(OVX)-induced osteoporotic mice model through an improved proliferation and differentiation of osteoblast cells [11]. In addition, another study has demonstrated that AJ enhanced anti-osteoporotic effects in osteoporosis-induced ovariectomized rats through increased bone alkaline phosphatase levels [12]. Despite the remarkable anti-osteoporotic effects of these two herbal plants, there have been no reports regarding the combination of LRC and AJ extracts for bone health.

In the present study, therefore, we aimed to investigate the alternative herbal therapeutic plants (LRC and AJ) for anti-osteoporotic effect in vivo and in vitro. This study examined osteoprotective effects of *Lycii radicis cortex* (LRC) and *Achyrantes japonica* (AJ) in osteoblast and osteoclast cells and ovariectomized mice.

## 2. Results

### 2.1. Lycii Radicis Cortex (LRC) and Achyrantes Japonica (AJ) Increased Osteoblast Differentiation and Mineralized Nodule Formation

**Figure 1:**
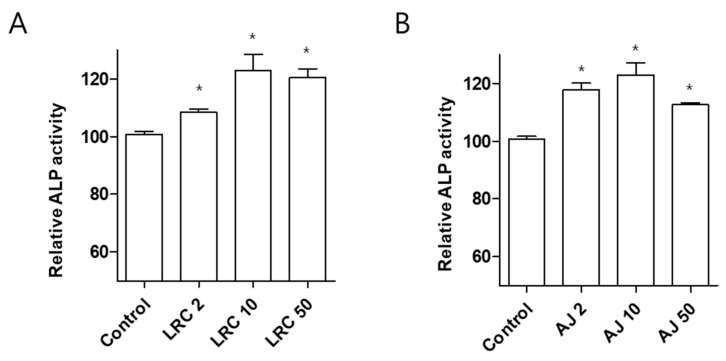
Effects of LRC and AJ extracts on cellular differentiation of the osteoblast-lineage cell line MC3T3-E1. Assessment of alkaline phosphatase (ALP) activity is shown for LRC (**A**) and AJ (**B**) extracts in MC3T3-E1 osteoblastic cells. After induction of osteoblast differentiation with 50 μg/mL of ascorbic acid and 10 mM of β-glycerophosphate, cells were cultured with three different concentrations (2, 10, and 50 μg/mL) for 3 days, and alkaline phosphatase (ALP) activity was analyzed. *: *p* < 0.05 vs. Control.

We investigated anti-osteoporotic effects of LRC and AJ extracts in osteoblastic cell lines MC3T3-E1. Pre-osteoblastic cells were treated with three different concentrations (2, 10, and 50 μg/mL) of LRC and AJ extracts for 3 days, and bone formation enhancing effects were assessed by ALP activity. Treatment of both LRC and AJ extracts significantly increased ALP activity but did not affect cell proliferation (Figure 1A,B and Figure S1). The highest ALP activity in the MC3T3-E1 cell line was detected in 10 μg/mL extracts of LRC and AJ.

**Figure 2:**
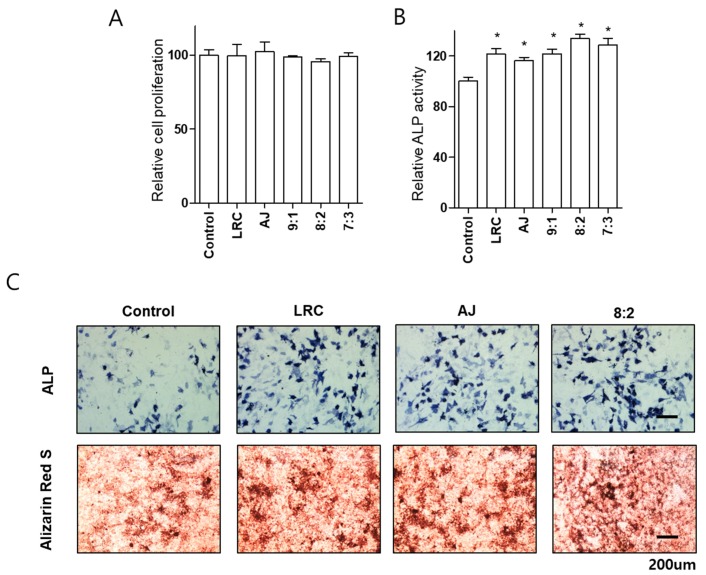
Effects of single or combined LRC and AJ extracts on cellular proliferation, differentiation, and mineralized nodule formation of the osteoblast-lineage cell lines. Assessment is shown for cell proliferation (**A**) and alkaline phosphatase (ALP) activity (**B**) of single or combined LRC and AJ (9:1, 8:2 or 7:3 ratio) in MC3T3-E1 cells. Osteoblast differentiation was induced by adding 50 μg/mL of ascorbic acid and 10 mM of β-glycerophosphate, MC3T3-E1 cells were incubated with 10 μg/mL of single or combined LRC and AJ extracts (9:1, 8:2 or 7:3) for 3 days, and then cellular proliferation and ALP activity were assessed. (**C**) ALP and Alizarin Red S staining was assessed in single or combined LRC and AJ-treated cells. Cells were treated with 10 μg/mL of single or combined LRC and AJ extracts (8:2) for 3 days (for ALP staining) or 21 days (for Alizarin Red S staining), and osteoclast differentiation and mineralized nodule formation were stained. Control: non-treated cells. *: *p* < 0.05 vs. Control.

To further confirm the synergistic effect of LRC and AJ extract on the cellular differentiation of osteoblasts, we tested ALP activity of combined LRC and AJ extracts. Osteoblastic cells were treated with 10 μg/mL single extracts of LRC and AJ and various combined LRC and AJ extract ratios (9:1, 8:2 or 7:3), and ALP activity was assessed at 2, 3, 4, and 5 days. Since LRC has been more frequently used for treatment osteoporosis in eastern Asia as a traditional medicine, we carried out a higher amount of LRC extract ratio, compared to AJ extract. Unlike a previous study reporting bone-enhancing effects of Korean LRC extracts [11], in the present study, Chinese LRC extracts were used for all the experiments. We found higher concentrations of scopolin in Chinese LRC than in Korean LRC plants (Figure S2). Noteworthy, a study has shown that scopolin has anti-osteoporotic effects by inhibiting the differentiation of osteoclastic macrophage RAW 264.7 cells [13]. The combination of LRC and AJ extracts did not affect cell proliferation (Figure 2A). Significantly increased ALP activity was observed in all extracts at 3 days incubation, compared to 2, 4, and 5 days treatment (Figure S3). The combined LRC and AJ 8:2 ratio showed the highest ALP level, and ALP positive staining colonies in MC3T3-E1 cells (Figure 2B,C).

Next, we examined the effects of LRC and AJ on mineralized nodule formation. Differentiated osteoblast cells are mineralized through the induction of calcium and phosphate-based minerals with the development of several matrix proteins [2]. We confirmed that the MC3T3-E1 cells were mineralized by induction reagents (ascorbic acid and β-glycerophosphate) for 21 days. Osteoblast cells were treated with single extracts of LRC and AJ and combined LRC and AJ 8:2 ratio at 10 μg/mL. Cells treated with a combination of LRC and AJ extracts presented higher positive alizarin red S staining colonies than the single extracts of LRC and AJ extracts (Figure 2C).

### 2.2. LRC and AJ Increased mRNA Expression of Osteoblastic Markers

**Figure 3:**
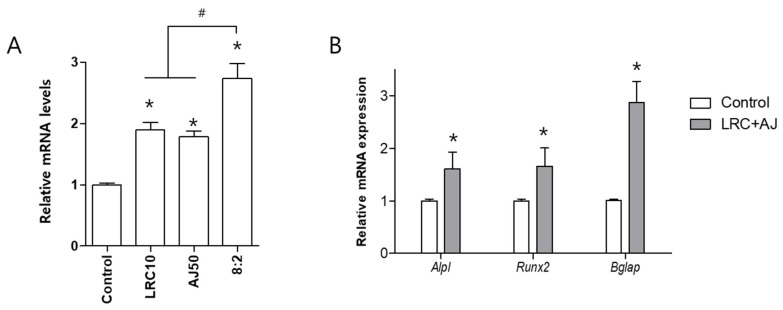
Effects of LRC and AJ extract on mRNA expression of bone formation markers (*Alpl*, *Runx2* and *Bglap*) *in* MC3T3-E1 cells. (**A**) Cells were treated with single extracts of LRC and AJ and combined LRC and AJ, with an 8:2 ratio at 10 μg/mL. Total RNA of the cells was extracted and then the mRNA expression level of *Bglap* was compared by quantitative reverse-transcription polymerase chain reaction (qRT-PCR). (**B**) The mRNA expression of *Alpl, Runx2* and *Bglap* was confirmed in the combined LRC and AJ extracts. The mRNA levels of bone formation markers were normalized by mRNA expression of **Gapdh** (glyceraldehyde 3-phosphate dehydrogenase). Control: non-treated cells. *: *p* < 0.05 vs. Control and #: *p* < 0.05 vs. 8:2.

We compared the expression of *Bglap* between single extracts of LRC and AJ and combined LRC and AJ in osteoblast cells (Figure S4). The combination of LRC and AJ extracts presented higher expression of *Bglap,* compared to single extracts of LRC and AJ (Figure 3A). Next, we confirmed the mRNA expression of *Alpl, Runx2* and *Bglap* in the combined LRC and AJ extracts. Significantly higher expression of *Alpl, Runx2* and *Bglap* was observed in LRC plus AJ-treated cells, compared to control (Figure 3B).

### 2.3. LRC and AJ Dcecreased Osteoclast Differentiation in Primary-Cultured Preosteoclast-Lineage Monocytes

**Figure 4:**
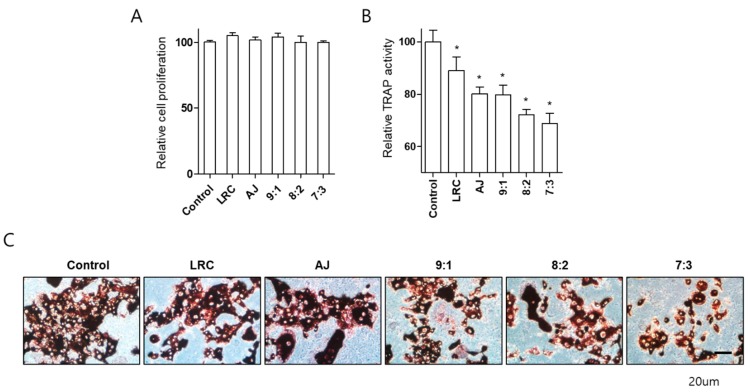
Effects of single or combined LRC and AJ extracts on cellular proliferation and differentiation of primary-cultured monocytes. Assessment were made of cell proliferation (**A**) and tartrate-resistant acid phosphatase (TRAP) activity in monocytes from mouse bone marrow (**B**). Osteoclast differentiation was induced by adding 30 ng/mL of M-CSF and 50 ng/mL of RANKL, and cells were treated with single or combined LRC and AJ (9:1, 8:2 or 7:3 ratio). The differentiated osteoclast cells were analyzed by TRAP activity (**B**) and TRAP staining (**C**). Control: Non-treated monocyte cells cultured with M-CSF and RANKL. *: *p* < 0.05 vs. Control.

We examined the effect of LRC and AJ extracts on differentiation of pre-osteoclast cells isolated from bone marrow in mice. Monocytes, osteoclast precursors, were effectively isolated from 6-week-old mice bone marrow and confirmed with monocyte-specific markers (CD11b antibody) using *fluorescence-activated cell sorting* analysis (Figure S5). Primary-cultured monocytes were induced by M-CSF and receptor activator of RANKL for osteoclast differentiation. After the induction of osteoclast differentiation, cells were treated with single or combined LRC and AJ extracts at 10 μg/mL for either 3 or 6 days, and osteoclast differentiation was analyzed by tartrate-resistant acid phosphatase (TRAP) activity (Figure S6). Extracts did not affect primary cultured monocyte proliferation during osteoclast differentiation (Figure 4A). LRC and AJ extracts significantly inhibited TRAP activity, and TRAP staining was positive for cells at 6 days of incubation, and combined LRC and AJ extracts more efficiently reduced osteoclast differentiation (Figure 4B,C).

### 2.4. LRC and AJ Inhibited OVX-Induced BMD Loss in Osteoporosis Model Mice

**Figure 5:**
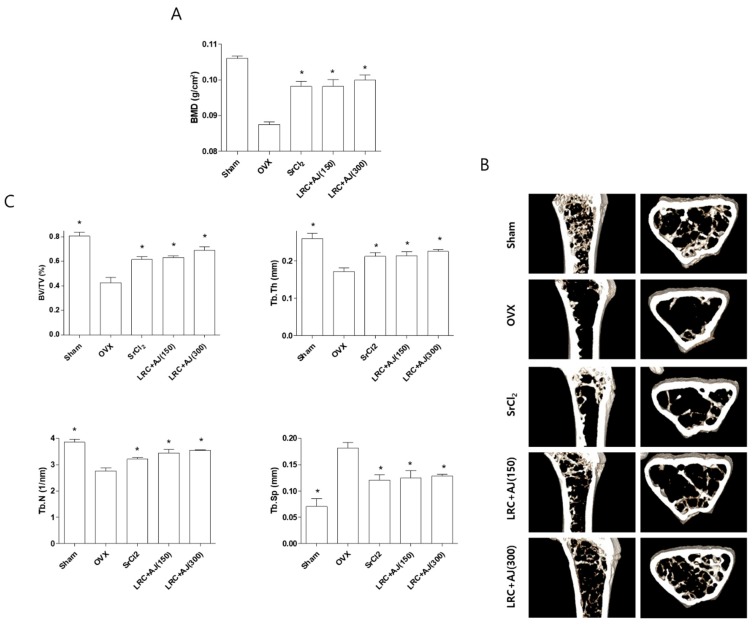
Anti-osteoporotic effects of LRC+AJ extract on the improvement of bone mineral density (BMD) and bone structural properties in OVX mice. The OVX mice were administered with either SrCl_2_ (10 mg/kg/day) or LRC+AJ extract (150 or 300 mg/kg/day) for 12 weeks. (**A**) BMD of the right femur was measured using a PIXI-mus bone densitometer. (**B**) Transverse micro-CT images were scanned using microcomputed tomography (CT). (**C**) Trabecular bone structural properties of bone volume (BV/TV), trabecular thickness (Tb.Th), number (Tb.N) and spacing (Tb.Sp) were analyzed at the end of the experiment. Sham: sham operated, OVX: non-Scopolin-administered mice. *: *p* < 0.05 vs. OVX group.

Based on an in vitro study design, we further examined the anti-osteoporotic effects of LRC+AJ extract on OVX-induced bone loss in an animal model. Eight-week-old female ddY mice were sham-operated (*n* = 6) or ovariectomized (*n* = 24) and divided into five groups: (1) Sham, (2) OVX control, (3) OVX administrated with 10 mg/kg/day of strontium chloride (SrCl_2_) as a positive control for bone formation, (4) OVX administrated with 150 mg/kg/day of LRC+AJ extract, and (5) OVX administrated with 300 mg/kg/day of LRC+AJ extract. In vivo toxic effects were evaluated using non-surgery mice with a mixture of LRC+AJ (150 and 300 mg/kg/day) for 12 weeks (Figure S7), and food intake and body weight did not differ between OVX and LRC+AJ-treated groups (data not shown). At the end of the experiment, BMD and BMC of the right femur were measured using a PIXI-mus bone densitometer, and transverse micro-CT images were scanned using a microcomputed tomography (CT) to analyze bone volume (BV/TV), trabecular thickness (Tb.Th), number (Tb.N) and spacing (Tb.Sp). Significant trabecular bone loss was found in the OVX group, compared to the sham group. As expected, positive control SrCl_2_ treatment for 12 weeks prevented OVX-induced BMD loss when measuring trabecular bone structural properties such as BV/TV, Tb.Th, Tb.N, and Tb.Sp. compared to the OVX group (Figure 5A–C), but BMC did not differ among OVX, SrCl_2_ and LRC+AJ-treated groups (data not shown). Similarly, the treatment of LRC+AJ extract (150 and 300 mg/kg/day) inhibited BMD loss and improved BV/TV, Tb.Th, Tb.N, and Tb.Sp with scanned micro-CT mages of the right femur in the osteoporotic animal model, compared to the OVX control group (Figure 5A–C).

**Figure 6:**
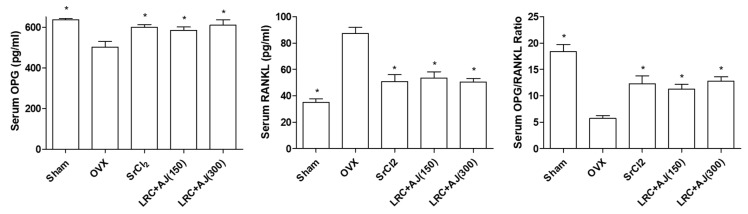
Effects of LRC+AJ extract on serum levels of bone metabolic makers (osteoprotegrin (OPG) and Receptor Activator of Nuclear Factor kappa-B ligand (RANKL)). After 12 weeks of administration, the blood samples were collected and serum levels of OPG and RANKL were measured using ELISA. *: *p* < 0.05 vs. OVX control.

To confirm the anti-osteoporotic effects of LRC+AJ extract on serum levels of bone metabolic makers, we collected the blood samples at the last day of treatment, and bone metabolic makers (osteoprotegrin (OPG) and Receptor Activator of Nuclear Factor kappa-B ligand (RANKL)) were measured using ELISA (enzyme-linked immunosorbent assay). The osteoporotic OVX model showed increased RANKL and decreased OPG but LRC+AJ extract inhibited bone loss through the suppression of bone resorption. Consequently, the treatment of LRC+AJ extract increased the ratio of OPG/RNAKL (Figure 6).

## 3. Discussion

Bone remodeling results from the balanced action of bone formation by osteoblasts and bone resorption by osteoclasts, to maintain calcium homeostasis and repair damaged bones [2,14]. Bone formation is regulated by various factors, such as collagen synthesis, mineralization, osteoblastic proliferation, and ALP activity [15]. Bone resorption is regulated by osteoclastic proliferation and differentiation and by the activation of TRAP [16]. However, an imbalance between these two processes leads to abnormal metabolic bone diseases, such as osteoporosis [17]. The present study describes the anti-osteoporotic effects of LRC and AJ in osteoblast and osteoclast cell cultures as well as in ovariectomized mice.

We first investigated the effects of LRC and AJ extracts on osteoblastic differentiation in MC3T3-E1 cells. ALP is a membrane-bound tetrameric enzyme found in the plasma membrane of osteoblast cells; it is considered as a reliable indicator of bone metabolism. In addition, ALP activity plays a crucial role in osteoid formation and mineralization, increasing its levels during osteoblast differentiation [18,19]. Hence, we used an ALP activity assay as a main method for determining the anti-osteoporotic effects of LRC and AJ. In addition, it is known that mineralized nodule formation is a common method for evaluating the effects of drug tests on bone matrix and bone formation [20,21]. Alizarin red S has been used for testing for calcium-rich deposits in mineralized osteoblast cells; positive alizarin red S staining represent successful mineralization of osteoblast cells in vitro [22]. Moreover, previous studies revealed that both LRC and AJ extracts improved anti-osteoporotic effects in ovariectomized-induced osteoporotic animal models [11,12]. In the present study, treating MC3T3-E1 cells with individual LRC and AJ extracts significantly promoted osteoblast differentiation, whereas a combined LRC and AJ treatment more effectively enhanced osteoblast differentiation and mineralization.

It has been shown that cellular differentiation of osteoblasts is regulated by several bone remodeling marker genes, such as *Alpl* (alkaline phosphatase, ALP), *Runx2* (runt-related transcription factor 2, Runx2), and *Bglap* (bone gamma carboxyglutamate protein, Osteocalcin) [18,23,24]. ALP concentration elevates with the induction of osteoblast differentiation leading to an active formation of new bones [18]. Runx2 is an essential transcription factor needed for proper osteoblast differentiation; its expression is upregulated in immature osteoblast cells [23]. Additionally, osteocalcin is secreted from osteoblasts and plays an important role in the regulation of bone metabolism [24]. These studies suggest that osteoblast differentiation is associated with a high expression of these bone metabolic markers. We found that LRC and AJ extracts increased mRNA expression of *Alpl*, *Runx2*, and *Bglap* during osteoblast differentiation, suggesting that LRC and AJ promoted osteoblast cellular differentiation and mineralization by up-regulating these metabolic markers.

Imbalanced processes between bone resorption and bone formation cause abnormal bone metabolic diseases such as osteoporosis [1]. Postmenopausal osteoporosis results from decreased estrogen concentration, mainly leading to elevated bone resorption rather than to impaired bone formation, increasing bone fragility and the risk of fractures [25]. Osteoclasts are exclusive bone resorptive multinucleated cells differentiated by the mononuclear cells of the monocyte/macrophage family and stimulated by the monocyte/macrophage colony-stimulating factor (M-CSF) and the receptor activator of nuclear factor κB ligand (RANKL) [26]. Mononuclear cells of the monocyte lineage in the bone marrow have been considered to be precursors of osteoclast cells [27]. Our results showed that LRC and AJ extracts decreased both TRAP activity and positive cell TRAP staining, indicating that LRC and AJ provoked a decrease in osteoclast differentiation of monocytes isolated from mouse bone marrow.

OVX animal models are widely used in the investigation of osteoporosis and in testing new osteoporotic therapies [28]. Typically, osteoporosis progression is characterized by a decrease in bone mass and a deterioration of bone tissues. OVX female mice showed BMD loss as well as low quality trabecular bone structural properties. However, LRC and AJ treatment prevented osteoporosis-induced bone loss and repaired bone structural properties related to BV/TV, Tb.Th, Tb.N, and Tb.Sp. Serum levels of bone metabolic makers OPG and RANKL are critical mediators in bone metabolism. OPG is a cytokine receptor, also known as osteoclastogenesis inhibitory factor, that plays an important role in the regulation of bone metabolism [29]. RANKL is an osteoclast differentiation factor that is critically involved in bone resorption [30]. OPG is a decoy receptor for RANKL that inhibits RANK–RANKL interactions, resulting in the suppression of osteoclastogenesis [27]. OVX mice conditions stimulated activation of bone resorption, whereas LRC+AJ extract inhibited activation of osteoclastogenesis through the induction of an increased OPG/RANKL ratio. These results suggested that the LRC+AJ extract prevented OVX-induced trabecular bone loss and osteoclastogenesis in an osteoporotic mouse model.

## 4. Materials and Methods

### 4.1. Cell Culture and Reagents

A mouse MC3T3-E1 cell line was purchased from the RIKEN Cell Bank (Tsukuba, Japan). The cells were cultured in Dulbecco’s modified Eagle’s (DMEM) medium supplemented with 10% FBS, penicillin (100 U/mL) and streptomycin (100 μg/mL). For osteoblast differentiation, pre-osteoblast cells were induced by adding β-glycerophosphate (10 mM) and an osteogenic medium containing ascorbic acid (50 μg/mL). The medium was changed every 3 days. All cultured cells were incubated in a humidified atmosphere at 37 °C and at 5% CO_2_.

### 4.2. Cell Proliferation Assay

The cells were incubated in a 96-well plate overnight and co-treated with CO and AJ extracts in the medium for 48 h. Cell proliferation was measured by water-soluble tetrazolium salt (WST) assay using an EZ-Cytox Cell Viability Assay Kit (Daeil; Seoul, Korea). Cells were incubated with WST solution (20 µL, 5 mg/mL in phosphate-buffered saline, PBS) for 4h. After incubation, absorbance values were measured at 450 and 655 nm using a microplate reader (BioTek, Winooski, VT, USA).

### 4.3. Alkaline Phosphatase (ALP) Assay and Staining

After induction of osteoblast differentiation, ALP activity and staining was measured in total cell lysates at 4 °C in a buffer containing 1 mmol/L Tris–HCl (pH 8.8), 0.5% Triton X-100, 10 mmol/L Mg^2+^, and 5 mmol/L p-nitrophenylphosphate. After homogenization, absorbance values of ALP activity were measured at 405 nm (BIO-RAD; Hercules, CA, USA). For ALP staining, cells were fixed in cold 4% paraformaldehyde and stained with a BCIP/NBT (Sigma-Aldrich; St. Louis, MO, USA). ALP stained cells were determined under a light microscope.

### 4.4. Mineralized Nodule Formation

Mineralized nodule formation in osteoblastic MC3T3-E1 cells was induced by adding 50 μg/mL of ascorbic acid and 10 mM of β-glycerophosphate for 21 days. After induction, cells were fixed in cold 4% paraformaldehyde and calcium deposits of the mineralized osteoblast cells were stained with Alizarin red S (Sigma-Aldrich; St. Louis, MO, USA). Positive stained cells were visualized using a light microscope.

### 4.5. Quantitative Reverse-Transcription PCR (qRT-PCR)

Total RNA extraction from cultured cells was performed using TRIzol reagent (Invitrogen; Carlsbad, CA, USA) following the manufacturer’s instructions. Complementary DNA(cDNA) from extracted RNA was synthesized via being reverse transcribed using a RevertAid™ H Minus First Strand cDNA Synthesis Kit (Fermentas; Hanover, NH, USA). Real-time reverse transcription polymerase chain reaction (RT-PCR) amplifications were performed using the ABI Prism 7000 Sequence Detection System (Applied Biosystems; Foster City, CA, USA), in a total volume of 25 μL containing 150 ng of cDNA using an SYBR Green I qPCR Kit (TaKaRa; Shiga, Japan). The specific primers for bone remodeling markers were as follows: 5′-TCC CAC GTT TTC ACA TTC GG-3′ and 5′-GGC CAT CCT ATA TGG TAA CGG G-3′ for mouse *Alpl*, 5′-TAAAGTGACAGTGGACGG TCCC-3′ and 5′-CCTCAGTGATTTAGGGCGCA-3′ for mouse *Runx2,* 5′-ATG GCG TCC TCT CTG CTT G-3′, 5′-TGA AAG GTC AGC GTA TGG CTT-3′ for mouse *sp7*, 5′-TAG TGA ACA GAC TCC GGC GCT A-3′ and 5′-ATG GCT TGA AGA CCG CCT ACA-3′ for mouse *Bglap,* and 5′-TGA CCA CAG TCC ATG CCA TC-3′ and 5′-GAC GGA CAC ATT GGG GGT AG-3′ for mouse *Gapdh*. The relative quantification of gene expression was normalized to the expression of **Gapdh** and analyzed using the comparative threshold (*C*t) method (Applied Biosystems). Relative gene expression was presented as 2^−Δ*C*t^ (Δ*C*t = *C*t _target gene_ − *C*t _Gapdh_) and the fold change was analyzed as 2^−ΔΔ*C*t^ (ΔΔ*C*t = Δ*C*t _control_ − *C*t _treatment_).

### 4.6. In Vitro Generation of Osteoclasts

The primary culture of monocytes was isolated from bone marrow cells of the femoral bones of 6-week-old mice with a fine-bore syringe. The identification of monocyte cells from bone marrow cells was performed by immunophenotypic analysis of monocyte-specific antibody CD11b (BioLegend; San Diego, CA, USA). Osteoclast differentiation form-isolated monocyte cells were induced by adding 30 ng/mL of M-CSF (PeproTech; Rocky Hill, CT, USA) and 50 ng/mL of RANKL (PeproTech) in an α-MEM medium [31]. After the induction of the monocytes, differentiated osteoclast cells were measured by a tartrate-resistant acid phosphatase (TRAP) activity and staining using an Acid-Phosphatase Kit (Sigma-Aldrich; St. Louis, MO, USA), following the manufacturer’s instructions.

### 4.7. Ovariectomized Model

The 8-week-old female ddY mice (ovariectomized (OVX, *n* = 24) and sham-operated (Sham, *n* = 6) were purchased from Shizuoka Laboratory Center Inc. (Hamamatsu, Japan). Mice were acclimated for 2 weeks before the in vivo experiment. Mice were maintained individually in clear plastic cages on a diet (5.0 g/day) of Formula-M07 (Feedlab Co., Ltd., Hanam, Korea) and tap water (15 mL/day), under control of temperature (23 ± 2 °C), humidity (55 ± 5%), and illumination (12-h light/dark cycle). Mice were treated with combined CO+RF extract (150 or 300 mg/kg/day) for 12 weeks. All experiments were conducted in accordance with the institutional guidelines established by the Committee of the Ajou University School of Medicine (AMC-133).

### 4.8. Measurement of Bone Loss in OVX Mice

At the end of the animal experiment, the mice were anesthetized using tiletamine/zolazepam (Zoletil; Virbac Laboratories, Carros, France), and right femur bone mineral density (BMD) and bone mineral content (BMC) were measured using on-board PIXI-mus densitometer software (GE Lunar, Madison, WI, USA). Next, the right femur samples were removed and transverse micro-CT images were obtained by scanning using a micro-CT in the GSTEP (INVEON, SIEMENS, Germany). Two-dimensional axial and 3D images were reconstructed for qualitative and quantitative analyses, using Inveon Research Workplace and COBRA_Exxim (SIEMNS, Germany).

### 4.9. Blood Sampling and Serum OPG and RANKL Measurement

Blood samples were collected from the left ventricle of anesthetized rats into heparinized syringes. Plasma was separated by centrifugation at 1200× *g* for 15 min at 4 °C and assayed for bone metabolic makers (osteoprotegrin (OPG) and Receptor Activator of Nuclear Factor kappa-B ligand (RANKL)), using multiplex assays analyzed with Luminex (Merck Millpore, Burlington, MA, USA), according to the manufacturer’s instructions. The ratio of OPG and RANKL (OPG/RANKL) was used as a measure to describe the process of bone formation coupled with bone resorption.

### 4.10. Statistical Analysis

A statistical software package (SPSS 11.0 for Windows, SPSS Inc., Chicago, IL, USA) was used for performing the statistical tests. The statistical significance of differences was assessed by the Student′s *t*-test. A value of *p* < 0.05 was considered significant. The results were expressed as mean ± SEM.

## 5. Conclusions

This is the first study to examine the anti-osteoporotic effects of alternative herbal medicines such as the LRC+AJ extract, both in vitro and in vivo. The LRC and AJ extract at an 8:2 ratio effectively promoted osteoblast differentiation and mineralized bone formation via up-regulation of bone metabolic markers (*Alpl, Runx2* and *Bglap*). The LRC+AJ extract prevented OVX-induced BMD loss and improved trabecular bone structural properties in the osteoporotic mouse model through the inhibition of osteoclastogenesis. The results suggest that the LRC+AJ extract may be a potential candidate for the treatment and prevention of osteoporotic bone loss.

## Supplementary Materials

The following are available online at https://www.mdpi.com/2072-6643/11/11/2716/s1, Figure S1. Effects of LRC and AJ extracts on cell proliferation in preosteoblast MC3T3-E1 cells. Cells were treated with ascorbic acid (50 μg/mL) and β-glycerophosphate (10 mM) and cultured with three different concentrations (2, 10 and 50 μg/mL) and cell proliferation was assessed by WST; Figure S2. Effect of LRC extracts from Korean (KOR) or Chinese (CN) plants on osteoclast differentiation of primary-cultured monocytes. Fold change in scopolin concentration; Figure S3. Effects of single or combined LRC and AJ extract on alkaline phosphatase (ALP) activity in the osteoblast-lineage cell lines. Cells were treated with ascorbic acid (50 μg/mL) and β-glycerophosphate (10 mM) and incubated with 10 μg/mL of single or combined LRC and AJ extracts (9:1, 8:2 or 7:3), and an ALP activity assay was assessed at 2, 3, 4, and 5 days. Control: non-treated cells. *: *p* < 0.05 vs. control; Figure S4. Effects of single or combined LRC and AJ extract on mRNA expression of *Bglap.* Cells were treated with single extracts of LRC and AJ and combined LRC and AJ. The mRNA expression of *Bglap* was measured by RT-PCR. Control: non-treated cells. *: *p* < 0.05 vs. Control; Figure S5. The validation of the successful isolation of monocytes from mouse bone marrow. Primary-cultured monocytes were identified by an immunophenotypic analysis with a monocyte-specific surface positive marker (PE-conjugated CD11b antibody) using a *fluorescence-activated cell sorting* analysis; Figure S6. Effects of single or combined LRC and AJ extract on osteoclast differentiation of primary-cultured monocytes. After induction of osteoclast differentiation by treatment of 30 ng/mL of M-CSF and 50 ng/mL of RANKL (Induction), cells were co-treated with single extracts of LRC and AJ and combined LRC and AJ for 3 and 6 days, and tartrate-resistant acid phosphatase (TRAP) activity was assessed. *: *p* < 0.05 vs. Induction; Figure S7. Changes of total body weight for 12 weeks in non-surgery mice
