# Supplementary material for: Anti-Osteoporotic Effects of Combined Extract of Lycii Radicis Cortex and Achyranthes japonica in Osteoblast and Osteoclast Cells and Ovariectomized Mice
Source: Nutrients. 2019 Nov 9;11(11):2716. doi: 10.3390/nu11112716 (PMC6893723; doi:10.3390/nu11112716)
Supplement: Supplementary file 1 [file nutrients-11-02716-s001.pdf]

Anti-Osteoporotic Effects of combined extract of Lycii Radicis Cortex and Achyranthes japonica in Osteoblast and Osteoclast Cells and Ovariectomized Mice

Eunkuk Park, Jeonghyun Kim, Subin Yeo, Chun Whan Choi, Sang Woo Lee, Wan Yi Li, Ji-Won Lee, Jin-Hyok Park, Dam Huh and Seon-Yong Jeong

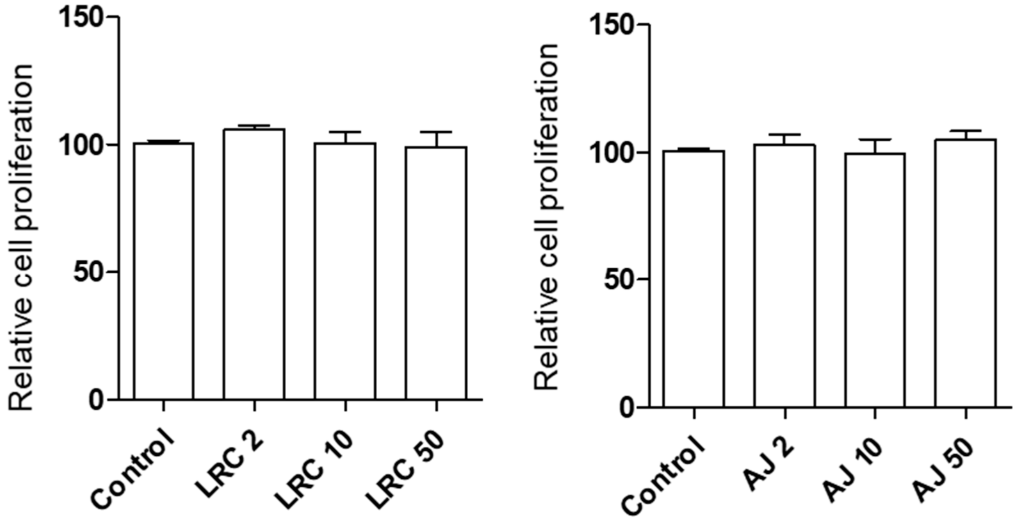

Figure. S1. Effects of LRC and AJ extracts on cell proliferation in preosteoblast MC3T3-E1 cells. Cells were treated with ascorbic acid (50 µg/ml) and β-glycerophosphate (10 mM) and cultured with three different concentrations (2, 10 and 50 µg/ml) and cell proliferation was assessed by WST.

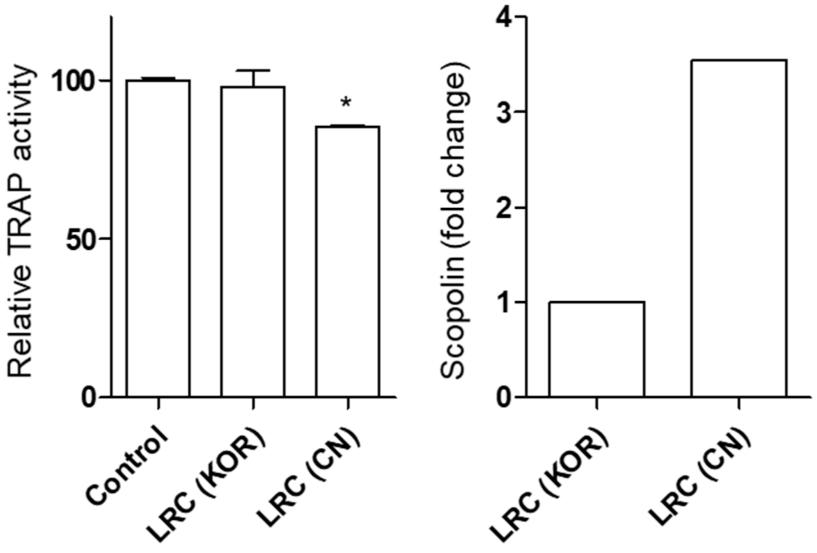

Figure. S2. Effect of LRC extracts from Korean (KOR) or Chinese (CN) plants on osteoclast differentiation of primary-cultured monocytes. Fold change in scopolin concentration.

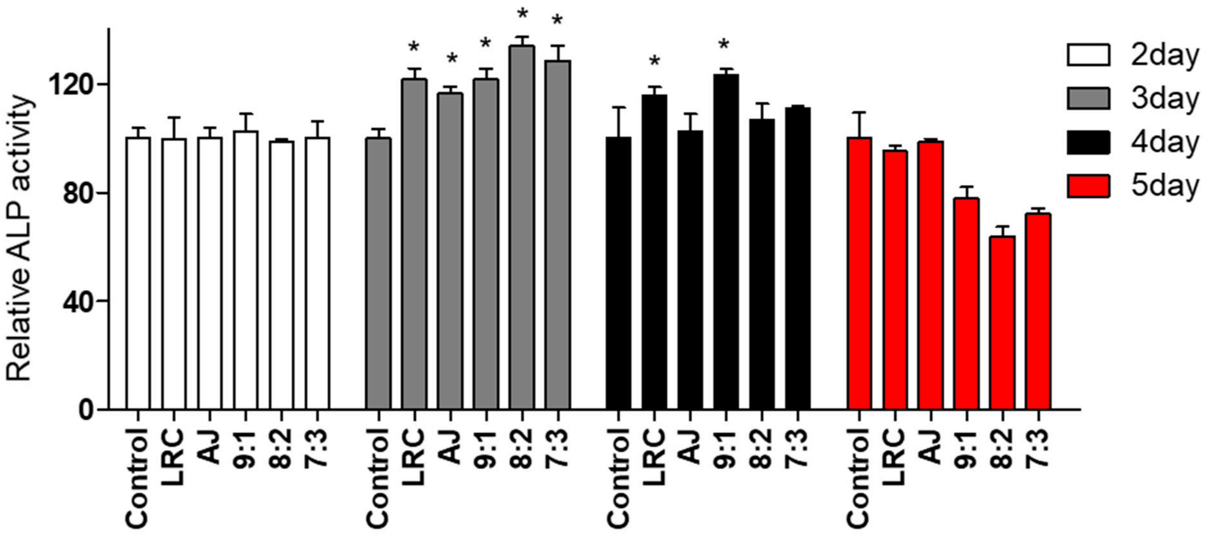

Figure. S3. Effects of single or combined LRC and AJ extract on alkaline phosphatase (ALP) activity in the osteoblast-lineage cell lines. Cells were treated with ascorbic acid (50 μg/ml) and β-glycerophosphate (10 mM) and incubated with 10 μg/ml of single or combined LRC and AJ extracts (9:1, 8:2 or 7:3), and an ALP activity assay was assessed at 2, 3, 4, and 5 days. Control: non-treated cells. *: $p < 0.05$ vs. control.

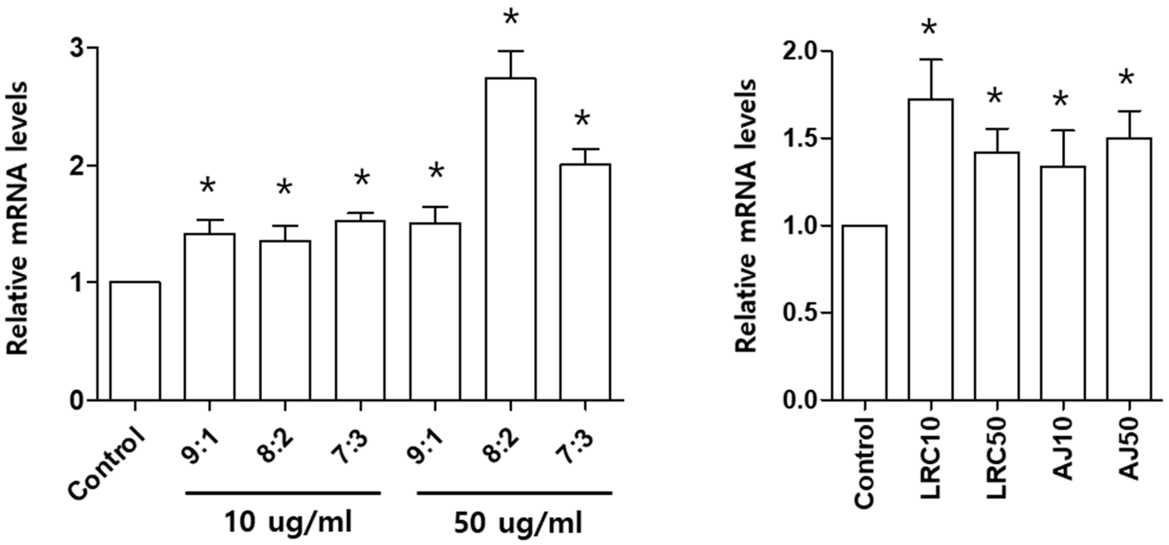

Figure. S4. Effects of single or combined LRC and AJ extract on mRNA expression of *Bglap*. Cells were treated with single extracts of LRC and AJ and combined LRC and AJ. The mRNA expression of *Bglap* was measured by RT-PCR. Control: non-treated cells. *: p<0.05 vs. Control.

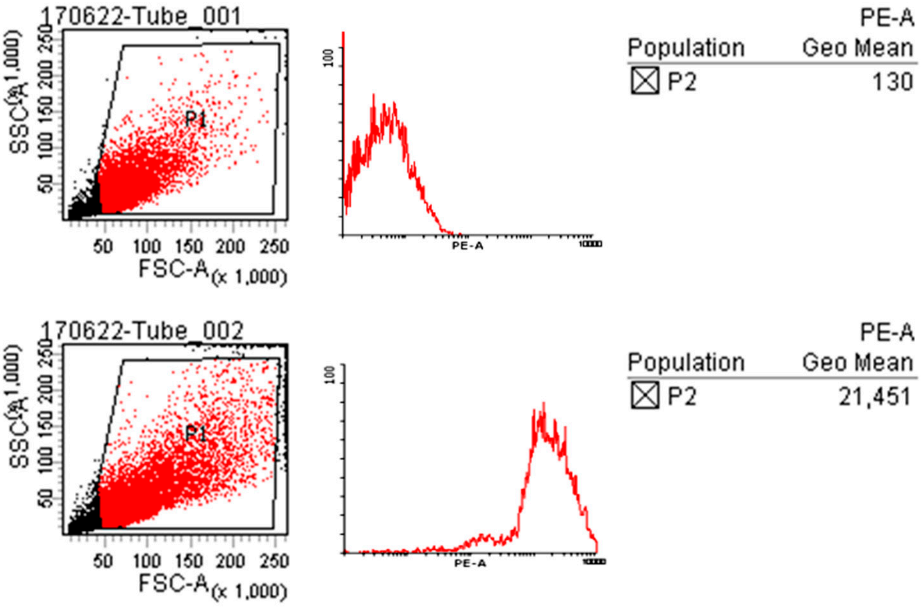

Figure. S5. The validation of the successful isolation of monocytes from mouse bone marrow. Primary-cultured monocytes were identified by an immunophenotypic analysis with a monocyte-specific surface positive marker (PE-conjugated CD11b antibody) using a fluorescence-activated cell sorting analysis.

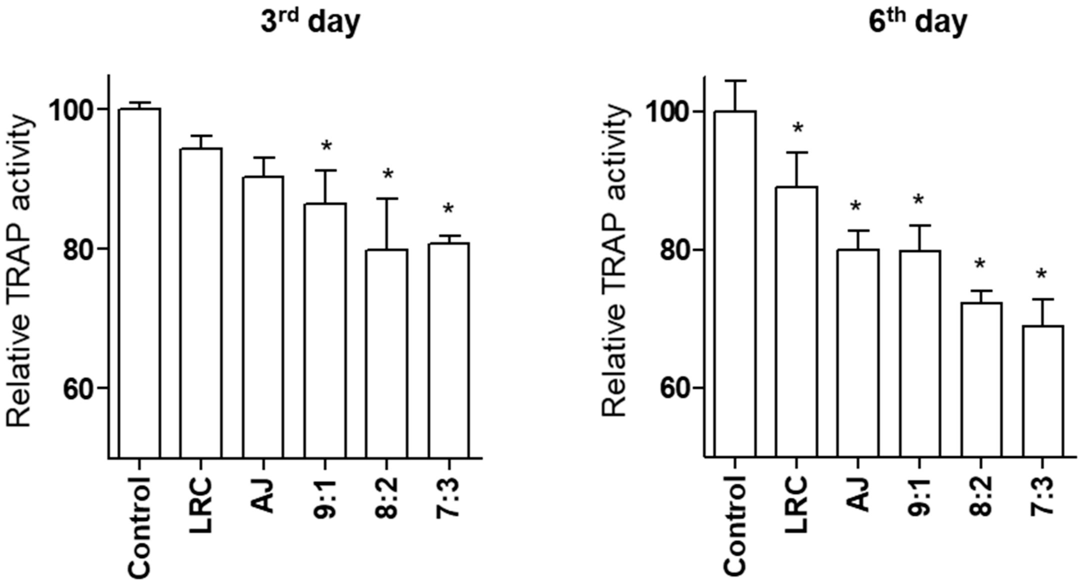

Figure. S6. Effects of single or combined LRC and AJ extract on osteoclast differentiation of primary-cultured monocytes. After induction of osteoclast differentiation by treatment of 30 ng/ml of M-CSF and 50 ng/ml of RANKL (Induction), cells were co-treated with single extracts of LRC and AJ and combined LRC and AJ for 3 and 6 days, and tartrate-resistant acid phosphatase (TRAP) activity was assessed. *: $p < 0.05$ vs. Induction.

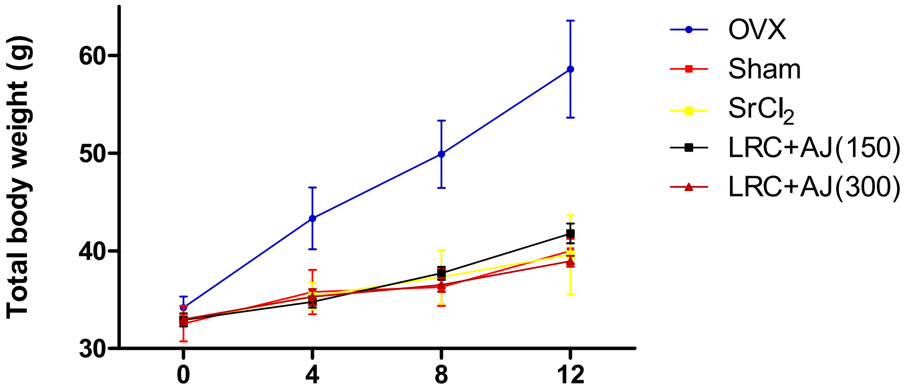

Figure. S7. Changes of total body weight for 12 weeks in non-surgery mice
